# Use of Common Psychiatric Medications and Risk and Prognosis of Amyotrophic Lateral Sclerosis

**DOI:** 10.1001/jamanetworkopen.2025.14437

**Published:** 2025-06-04

**Authors:** Charilaos Chourpiliadis, Anikó Lovik, Caroline Ingre, Rayomand Press, Kristin Samuelsson, Unnur Valdimarsdottir, Fang Fang

**Affiliations:** 1 Institute of Environmental Medicine, Karolinska Institutet, Stockholm, Sweden; 2 Institute of Psychology, Leiden University, Leiden, the Netherlands; 3 Department of Clinical Neuroscience, Karolinska Institutet, Stockholm, Sweden; 4 Department of Neurology, Karolinska University Hospital, Stockholm, Sweden; 5 Centre of Public Health Sciences, Faculty of Medicine, School of Health Sciences, University of Iceland, Reykjavik, Iceland; 6 Department of Epidemiology, Harvard T.H. Chan School of Public Health, Harvard University, Boston, Massachusetts

## Abstract

**Importance:**

Although several studies have shown an increased risk of subsequent amyotrophic lateral sclerosis (ALS) diagnosis for individuals with a history of psychiatric disorders, the evidence of an association between use of common psychiatric medications and ALS is scarce and inconclusive.

**Objective:**

To examine whether there is an association of prescribed use of common psychiatric medications, namely anxiolytics, hypnotics and sedatives, and antidepressants, with the risk and disease progression of ALS.

**Design, Setting, and Participants:**

This nationwide register-based case-control study was conducted in Sweden among all patients diagnosed with ALS from January 1, 2015, to July 1, 2023, according to the Swedish Motor Neuron Disease Quality Registry, who were age- and sex-matched with as many as 5 individuals with no ALS as well as their full siblings and spouses. Patients with ALS were followed up for a median (IQR) of 1.33 (0.64-2.37) years after diagnosis.

**Exposures:**

At least 2 prescriptions of the studied psychiatric medications before ALS diagnosis.

**Main Outcomes and Measures:**

The risk of ALS diagnosis associated with prediagnostic prescribed use of common psychiatric medications was estimated using conditional logistic regression models, comparing patients with ALS with population or relative control participants. Patients with ALS were followed up from diagnosis to assess the association of prediagnostic prescribed use of common psychiatric medications with disease progression. The association of mortality (or use of invasive ventilation) with the use of common psychiatric medications was estimated with a joint longitudinal-survival model accounting for the longitudinal changes of ALS Functional Rating Scale–Revised (ALSFRS-R) in the time-to-event analysis.

**Results:**

Among the 1057 case participants and 5281 population control participants, the mean (SD) age at diagnosis of the case participants (ie, date of selection of the control participants) was 67.5 (11.5) years, and 3363 (53.1%) were male. In the population comparison, prescribed use of common psychiatric medications across all studied time windows before ALS diagnosis was associated with a higher risk of ALS (eg, among individuals prescribed hypnotics and sedatives 0-1 year before diagnosis: odds ratio [OR], 6.10; 95% CI, 3.77-9.88; prescribed anxiolytics 1-5 years before diagnosis: OR, 1.60; 95% CI, 1.15-2.23; prescribed antidepressants >5 years before diagnosis: OR, 1.21; 95% CI, 1.02-1.44). Excluding the year before diagnosis from the analysis, prescribed use of anxiolytics (OR, 1.34; 95% CI, 1.12-1.60), hypnotics and sedatives (OR, 1.21; 95% CI, 1.02-1.43), or antidepressants (OR, 1.26; 95% CI, 1.06-1.49) was associated with an increased risk of ALS. Similar results were noted in the comparison with relative control participants, partially alleviating the concern on familial confounding, with the exception of hypnotics and sedatives. Shorter survival was demonstrated among patients with ALS who had prediagnostic use of anxiolytics (hazard ratio [HR], 1.52; 95% CI, 1.12-2.05) or antidepressants (HR, 1.72; 95% CI, 1.30-2.29), compared with patients with ALS without such experience.

**Conclusions and Relevance:**

In this case-control study, prescribed use of anxiolytics, hypnotics and sedatives, or antidepressants was associated with a higher subsequent risk of ALS. Prediagnostic use of such medications was also associated with a poor prognosis after ALS diagnosis.

## Key Points

**Question:**

Is there an association between prescribed use of common psychiatric medications and the risk and progression of amyotrophic lateral sclerosis (ALS)?

**Findings:**

In this case-control study including nearly 9000 individuals, prescribed use of anxiolytics, hypnotics and sedatives, or antidepressants was associated with a 34%, 21%, and 26% higher future risk of ALS. Prediagnostic use of such medications was also associated with poor prognosis after ALS diagnosis.

**Meaning:**

These findings suggest a potential link between psychiatric medications, or their indications (ie, psychiatric disorders), and the risk and progression of ALS.

## Teaser

This case-control study uses registry data from Sweden to evaluate whether the use of anxiolytics, hypnotics and sedatives, or antidepressants is associated with later diagnosis and progression of amyotrophic lateral sclerosis (ALS).

## Introduction

Amyotrophic lateral sclerosis (ALS) is a debilitating neurodegenerative disease, affecting the upper and lower motor neurons.^1,2^ Although ALS has been long considered a disease affecting only the motor neurons, it is increasingly recognized that patients with ALS manifest psychiatric symptoms.^3^ For instance, 11% and 30% of ALS patients have been shown to develop depression and generalized anxiety disorder, respectively.^4,5^ Considering the terminal nature of ALS, symptoms of depression and anxiety, especially soon after diagnosis, may constitute a psychological response.^6^ In addition, patients with psychiatric disorders have been shown to have an increased future risk for ALS.^7,8,9,10,11,12,13^ A higher-than-expected comorbidity between ALS and psychiatric disorders is biologically plausible. Shared genetic loci, such as *CNTN6*, encoding an axon structural protein, and *GLT8D1*, encoding a glycosyltransferase enzyme, indicate shared genetic risk between ALS and schizophrenia.^14,15^ Increased prevalence of schizophrenia and suicidal behavior among ALS kindreds suggests on the other hand that the pathogenic processes contributing to ALS might also contribute to the development of psychiatric disorders.^16^

Anxiolytics, hypnotics and sedatives, and antidepressants are the most frequently prescribed psychiatric medications.^17,18,19^ To our knowledge, few studies to date have used psychiatric medications as a proxy for psychiatric disorders to study their associations with the risk and progression of ALS.^10,12,20,21^ One study^20^ examined a comprehensive list of central nervous system (CNS) medications in relation to the risk of ALS, without including anxiolytics or hypnotics and sedatives. A few other studies examined the link between use of antidepressants and risk of ALS; however, they had conflicting results, partly due to varying study design (self-reported^10^ vs register-based^12^ data) or limited period in ascertaining medication use.^20^ To this end, we took advantage of nationwide health and population registers in Sweden, including the Swedish Motor Neuron Disease (MND) Quality Registry, to first explore the association between prescribed use of anxiolytics, hypnotics and sedatives, or antidepressants and the subsequent risk of ALS, after controlling for familial confounding, and to then explore the role of prediagnostic use of these medications in the survival and functional decline of patients with ALS.

## Methods

### Study Design

We performed a register-based nested case-control study. The Swedish MND Quality Registry has since 2015 collected data on clinical characteristics, patient-reported outcomes, and different biomarkers for more than 80% of MND patients in Sweden, including follow-up from diagnosis until death.^22^ From this registry, we identified a total of 1057 individuals with ALS (including definite, probable, and possible ALS according to the revised El Escorial Criteria) who were diagnosed between January 1, 2015, and July 1, 2023 (ie, case participants in the nested case-control study). Using the Swedish Total Population Register, we randomly selected 5 population control participants per case, who were individually matched to the case participant on age and sex and who were free of ALS until the case’s diagnosis date, using the method of incidence density sampling.^23^ Through the Total Population Register, we also identified all full siblings and spouses of the case participants. These individuals were free of ALS at the case participant’s diagnosis date and were considered relative control participants. Full siblings share, on average, 50% of their genetic factors and early-life environmental factors, whereas spouses share adult-life environmental factors. Therefore, comparisons with relative control participants could address potential familial confounding due to such shared factors.^24^ The date of ALS diagnosis was used as the index date for the case and their matched population or relative control participants. Finally, to understand the association between prediagnostic use of psychiatric medications and ALS prognosis, we performed a cohort study among the ALS patients with follow-up from date of diagnosis until death, initiation of invasive ventilation, emigration, or end of study (September 30, 2023), whichever came first.

The study was approved by the Swedish Ethical Review Authority. The requirement of informed consent was waived for this study as only registry-based secondary data were used. Reporting of the present study followed the Strengthening the Reporting of Observational Studies in Epidemiology (STROBE) reporting guidelines.

### Prescribed Use of Common Psychiatric Medications

We linked case and control participants to the Swedish Prescribed Drug Register using the unique Swedish personal identification numbers. Since July 1, 2005, this register has collected data on medications dispensed at all pharmacies in Sweden, coded according to the Anatomical Therapeutic Chemical (ATC) classification system.^25^ Through this linkage, we identified records of prescribed use of common psychiatric medications, namely anxiolytics, hypnotics and sedatives, and antidepressants, before the index date for the case and control participants, using the ATC codes N05B, N05C, and N06A, respectively. We identified prescribed use of these medications through at least 2 prescriptions to increase the specificity of the exposure definition. Through free text available in the Prescribed Drug Register, we also retrieved information on potential reasons for the prescription of these medications.

### Covariables

Information on date of birth and sex was accessed through the Total Population Register. Socioeconomic status, country of birth, and the highest educational attainment were also considered potential confounders^8,12^ and accessed through the Swedish Censuses during 1965 to 1990 and the Longitudinal Integrated Database for Health Insurance and Labor Market Studies (LISA) since 1990.^26^ Country of birth was classified as Sweden, other Nordic, European countries not including the Nordic (EU28), and non-European countries. Classification of socioeconomic status was based on the educational requirement of occupations, while the highest educational attainment was calculated based on the duration of a person’s formal education.

### Clinical Characteristics of Patients With ALS

Through the Swedish MND Quality Registry, we obtained data on clinical characteristics of the case participants at diagnosis and during follow-up. Site of disease onset was classified as bulbar, spinal, or other (including respiratory and frontal lobe onset). Delay in diagnosis was calculated as the time interval between onset of muscle paresis and diagnosis. Disease progression rate at diagnosis was calculated by the following formula: (48 – ALS Functional Rating Scale–Revised [ALSFRS-R] score at diagnosis)/diagnostic delay (in months). Body mass index (BMI; calculated as weight in kilograms divided by height in meters squared) at diagnosis, use of gastrostomy and invasive ventilation after diagnosis, and familial history of ALS were also considered. Finally, the functional decline of patients with ALS was calculated using the ALSFRS-R scores measured at diagnosis and follow-up visits (approximately every 6 months since diagnosis).

### Statistical Analysis

#### Risk of ALS Diagnosis

In the analysis of the nested case-control study, we used conditional logistic regression to examine the association of use of anxiolytics, hypnotics and sedatives, and antidepressants with the risk of ALS. First, we examined the association through comparing patients with ALS with population control participants. We then compared patients with ALS with the sibling and spouse control participants to examine the potential impact of familial confounding. We examined use of medications more than 5 years, 1 to 5 years, or 0 to 1 year before the index date, to understand the temporal pattern of the association. As the median diagnostic delay of ALS was about 1 year in the study sample, we then excluded the 1 year before the index date from the analysis to avoid reverse causation and performed stratified analyses by sex and age at the index date (<65 or ≥65 years). The conditional logistic regression models were automatically adjusted for sex, age, and calendar time at the index date, given the individually matched design and the use of incidence density sampling, and were further adjusted for educational attainment, socioeconomic status, and country of birth.

We performed several sensitivity analyses. First, to assess the robustness of our findings to the definition of exposure to psychiatric medications, we used at least 1 prescription to define the exposure and examined its association with the risk of ALS. Second, we performed a sensitivity analysis after additionally adjusting for history of psychiatric disorders at the time of exposure, as an effort to differentiate the impact of psychiatric medications from psychiatric disorders. History of psychiatric disorders was ascertained as any previous diagnosis of psychiatric disorders made at an inpatient (since 1973) or outpatient (since 2001) hospital visit. Finally, we separately analyzed the periods 1 to 2 years and 2 to 5 years before ALS diagnosis to provide a finer description of the temporal trend of the associations.

#### Mortality Risk and Functional Decline After an ALS Diagnosis

In the analysis of the cohort study of patients with ALS, we first compared the clinical characteristics of patients with ALS by their prediagnostic use of prescribed psychiatric medications. Second, we estimated the risk of death after an ALS diagnosis associated with prediagnostic use of psychiatric medications using a joint longitudinal-survival model that accounts for the longitudinal changes of ALSFRS-R since ALS diagnosis, to address the issue of informative censoring during the follow-up of patients with ALS.^27^ The joint model combines a longitudinal component with random intercept, slope, and unstructured covariance matrix and a Weibull survival component.^28^ The longitudinal and the survival components were linked through the shared random effects. Attained age was used as the underlying time scale. The model was adjusted for age at diagnosis, sex, BMI at diagnosis, diagnostic delay, disease progression rate at diagnosis, and site of onset. The proportionality assumption of the Weibull model used for the survival model was tested using Schoenfeld residuals, and an interaction with time was introduced for variables for which the assumption was violated. In addition to analyzing all patients with ALS as one group, we also performed stratified analyses by sex and age at diagnosis. To assess the importance of including the longitudinal component in this analysis, we performed a sensitivity analysis using Cox model only, after adjustment for age at diagnosis, sex, BMI at diagnosis, diagnostic delay, disease progression rate at diagnosis, site of onset, and ALSFRS-R score measured at diagnosis.

Third, we used a linear mixed model with random intercept and slope to study the mean change in ALSFRS-R score over time associated with prediagnostic prescribed use of psychiatric medications, to assess the association of such use with the rate of functional decline after ALS diagnosis. Time was measured in intervals of 6 months, whereas the SEs of changes were calculated using robust sandwich estimator.^29^ This analysis was adjusted for age at diagnosis, sex, BMI at diagnosis, diagnostic delay, site of onset, and ALSFRS-R score measured at diagnosis.

All analyses were conducted using Stata version 16 (StataCorp). A 2-tailed 5% level of significance was considered in all analyses. In all analyses, we performed a complete case analysis including patients with complete information on all variables.

## Results

In the nested case-control study including 1057 case participants and 5281 population control participants, the mean (SD) age at index date was 67.5 (11.5) years, and 3363 (53.1%) were male (Table 1). Spinal onset was observed in 363 case participants (34.3%). Patients with ALS had a mean (SD) BMI of 23.8 (4.3) and ALSFRS-R score of 36.1 (8.1) at diagnosis. The median (IQR) diagnostic delay was 11.7 (7.3-18.3) months. The specific reasons for prescriptions among patients with ALS more than 1 year before diagnosis are shown in eTable 10 in Supplement 1.

**Table 1.** Baseline Characteristics of Patients With ALS, Their ALS-Free Siblings and Spouses, and Age- and Sex-Matched Population Control Participants

Characteristics	Participants, No. (%)					
	Population		Sibling		Spouse	
	Cases (n = 1057)	Population controls (n = 5281)	Cases (n = 709)	Siblings (n = 1418)	Cases (n = 806)	Spouses (n = 871)
Age at index date, mean (SD), y	67.5 (11.5)	67.5 (11.5)	66.6 (11.2)	66.6 (12.4)	68.0 (11.2)	67.0 (12.4)
Sex						
Male	561 (53.1)	2802 (53.1)	386 (54.4)	713 (50.3)	434 (53.8)	393 (45.1)
Female	496 (46.9)	2479 (46.9)	323 (45.6)	705 (49.7)	372 (46.2)	478 (54.9)
Educational attainment						
<9 y	112 (10.6)	700 (13.3)	53 (7.5)	145 (10.2)	83 (10.3)	75 (8.6)
9-10 y	110 (10.4)	567 (10.7)	72 (10.2)	135 (9.5)	85 (10.6)	78 (9.0)
Upper secondary education	434 (41.1)	2277 (43.1)	302 (42.6)	605 (42.7)	328 (40.7)	359 (41.2)
Postsecondary <2 y	57 (5.4)	258 (4.9)	47 (6.6)	74 (5.2)	42 (5.2)	34 (3.9)
Postsecondary ≥2 y	317 (30.0)	1365 (25.9)	222 (31.3)	383 (27)	244 (30.3)	297 (34.1)
Postgraduate education	19 (1.8)	54 (1.0)	11 (1.6).	21 (1.5)	17 (2.1)	22 (2.5)
Missing	8 (0.8)	60 (1.1)	2 (0.3)	55 (3.9)	7 (0.9)	6 (0.7)
SES						
Occupation without educational requirement	48 (4.5)	318 (6.0)	31 (4.4)	65 (4.6)	31 (3.9)	21 (2.4)
Occupation requiring high school degree	457 (43.2)	2502 (47.4)	309 (43.5)	594 (41.9)	345 (42.8)	335 (38.5)
Occupation requiring university studies ≤3 y	188 (17.8)	788 (14.9)	140 (19.7)	198 (14)	153 (19)	146 (16.8)
Occupation requiring university studies >3 y	252 (23.8)	1052 (19.9)	182 (25.8)	326 (23)	200 (24.8)	258 (29.6)
Missing	112 (10.6)	621 (11.8)	47 (6.6)	235 (16.6)	77 (9.6)	111 (12.7)
Country of birth						
Sweden	916 (86.7)	4510 (85.4)	691 (97.5)	1370 (96.6)	692 (85.9)	726 (83.4)
Other Nordic country	45 (4.3)	206 (3.9)	9 (1.3)	23 (1.6)	35 (4.3)	38 (4.4)
EU28	51 (4.8)	275 (5.2)	8 (1.1)	17 (1.2)	42 (5.2)	52 (5.9)
Non-EU	45 (4.3)	290 (5.5)	1 (0.1)	8 (0.6)	37 (4.6)	55 (6.3)
Age at death, mean (SD)	70.4 (9.8)	NA	69.1 (9.5)	NA	70.7 (9.9)	NA
No.	768	NA	513	NA	597	NA
BMI at diagnosis, mean (SD)	23.8 (4.3)	NA	23.6 (4.1)	NA	23.9 (4.4)	NA
No.	773	NA	500	NA	587	NA
ALSFRS-R at diagnosis, mean (SD)	36.1 (8.1)	NA	36.8 (7.8)	NA	36.4 (7.9)	NA
No.	660	NA	435	NA	495	NA
Progression rate at diagnosis, median (IQR), points/mo	0.7 (0.4-1.2)	NA	0.6 (0.4-1.1)	NA	0.7 (0.4-1.2)	NA
No.	637	NA	419	NA	476	NA
Gastrostomy						
PEG	231 (21.9)	NA	147 (20.7)	NA	170 (21.1)	NA
RIG	7 (0.7)	NA	4 (0.6)	NA	5 (0.6)	NA
Wizelfistel	1 (0.1)	NA	1 (0.1)	NA	1 (0.1)	NA
None	818 (77.4)	NA	557 (78.6)	NA	630 (78.2)	NA
Invasive ventilation		NA		NA		NA
Yes	16 (1.5)	NA	11 (1.7)	NA	11 (1.4)	NA
No	1041 (98.5)	NA	698 (98.3)	NA	795 (98.6)	NA
Diagnostic delay, median (IQR), mo	11.7 (7.3- 18.3)	NA	12.2 (7.5- 19.1)	NA	11.9 (7.4- 17.2)	NA
No.	970	NA	710	NA	806	NA
Dementia						
Yes	74 (7)	NA	35 (4.9)	NA	63 (7.8)	NA
No	353 (33.4)	NA	232 (32.8)	NA	262 (32.5)	NA
Missing	630 (59.6)	NA	442 (62.3)	NA	481 (59.7)	NA
Familial ALS						
Sporadic	399 (37.8)	NA	251 (35.4)	NA	295 (36.6)	NA
Familial	49 (4.6)	NA	36 (5.2)	NA	38 (4.7)	NA
Missing	609 (57.6)	NA	422 (59.4)	NA	473 (58.7)	NA
Onset site						
Bulbar	230 (21.8)	NA	137 (19.3)	NA	173 (21.5)	NA
Spinal	363 (34.3)	NA	236 (33.4)	NA	270 (33.5)	NA
Other	45 (4.3)	NA	31 (4.4)	NA	35 (4.3)	NA
Missing	419 (39.6)	NA	305 (43)	NA	328 (40.7)	NA

Abbreviations: ALS, amyotrophic lateral sclerosis; ALSFRS-R, Amyotrophic Lateral Sclerosis Functional Rating Scale–Revised; BMI, body mass index (calculated as weight in kilograms divided by height in meters squared); EU28, European countries excluding the Nordic countries; NA, not applicable; PEG, percutaneous endoscopic gastrostomy; RIG, radiologically inserted gastrostomy; SES, socioeconomic status.

### Risk of ALS Diagnosis

In the population comparison, having at least 2 prescriptions of psychiatric medications was associated with a higher risk of ALS during 0 to 1 year, 1 to 5 years, or more than 5 years before ALS diagnosis, after multivariable adjustment (eg, among individuals prescribed hypnotics and sedatives 0-1 year before index date: odds ratio [OR], 6.10; 95% CI, 3.77-9.88; prescribed anxiolytics 1-5 years before index date: OR, 1.60; 95% CI, 1.15-2.23; prescribed antidepressants >5 years before index date: OR, 1.21; 95% CI, 1.02-1.44)(eTable 1 in Supplement 1). After excluding the year before index date from analysis, prescribed use of anxiolytics (OR 1.34; 95% CI 1.12-1.60), hypnotics and sedatives (OR 1.21; 95% CI 1.02-1.43), or antidepressants (OR 1.26; 95% CI 1.06-1.49) was associated with an increased risk of ALS (Table 2). The results were in general similar when comparing patients with ALS with the sibling or spouse control participants, although the association for hypnotics and sedatives was attenuated in the spouse comparison, and, to a lesser degree, in sibling comparison. These associations were consistently noted between male and female participants although the magnitude of the associations was greater among male than female participants; however, the associations were restricted to younger individuals (<65 years) and not noted among older individuals (≥65 years) (eTable 2 in Supplement 1). The same pattern by sex and age was also noted when focusing on prescribed use of psychiatric mediations more than 5 years before ALS diagnosis.

**Table 2.** Association Between Diagnosis of Amyotrophic Lateral Sclerosis and Prescribed Use of Anxiolytics, Hypnotics and Sedatives, or Antidepressants More Than 1 Year Before Diagnosis

Psychiatric medications	Comparison^a^					
	Population		Sibling		Spouse	
	No. of cases/No. of controls	OR (95% CI)	No. of cases/No. of controls	OR (95% CI)	No. of cases/No. of controls	OR (95% CI)
Anxiolytics	191/682	1.34 (1.12-1.60)	123/172	1.41 (1.06-1.86)	131/121	1.31 (0.98-1.76)
Hypnotics and sedatives	240/926	1.21 (1.02-1.43)	156/248	1.17 (0.90-1.51)	174/193	1.03 (0.78-1.36)
Antidepressants	235/898	1.26 (1.06-1.49)	150/248	1.15 (0.90-1.49)	171/163	1.35 (1.02-1.79)

Abbreviation: OR, odds ratio.

^a^ Conditioned on age, sex, and calendar time, and adjusted for socioeconomic status, educational attainment, and country of birth.

Similar associations were noted between prescribed use of psychiatric medications defined by at least 1 prescription and risk of ALS, although the magnitude of the association diminished to some extent (eTable 3 in Supplement 1). Further adjustment for history of psychiatric disorders did not change the results substantially (eTable 4 in Supplement 1). Finally, stronger associations were observed for individuals prescribed these medications 1 to 2 years before the index date, compared with 2 to 5 years, partly due to the small number of case participants exposed (eTable 5 in Supplement 1).

### Mortality Risk and Functional Decline After an ALS Diagnosis

Compared with patients without prediagnostic use of the studied psychiatric medications, those with such use were more likely to be female and had a lower ALSFRS-R score at diagnosis (eTable 6 in Supplement 1). Patients with ALS were followed up for a median (IQR) of 1.33 (0.64-2.37) years after diagnosis, during which we observed 1024 deaths or initiations of invasive ventilation. None of the patients with ALS were censored due to emigration. After diagnosis, patients with prediagnostic use of psychiatric medications had a higher risk of mortality consistently (Figure). After multivariable adjustment, prediagnostic use of anxiolytics (hazard ratio, 1.52; 95% CI, 1.12-2.05) or antidepressants (HR, 1.72; 95% CI, 1.30-2.29) was associated with a higher risk of mortality (Table 3). These associations were consistent across sex and age groups, although the associations were only consistently statistically significant for antidepressants (eTable 7 in Supplement 1). Cox model yielded similar results (eTable 8 in Supplement 1). Prediagnostic use of psychiatric medications was associated with a faster rate of decline in ALSFRS-R scores over time (Figure). After multivariable adjustment, the association was only statistically significant for antidepressants, with (β = −2.50; 95% CI, −4.44 to −0.57) or without (β = −2.48; 95% CI, −4.41 to −0.55) adjustment for ALSFRS-R at diagnosis (eTable 9 in Supplement 1).

**Figure.**
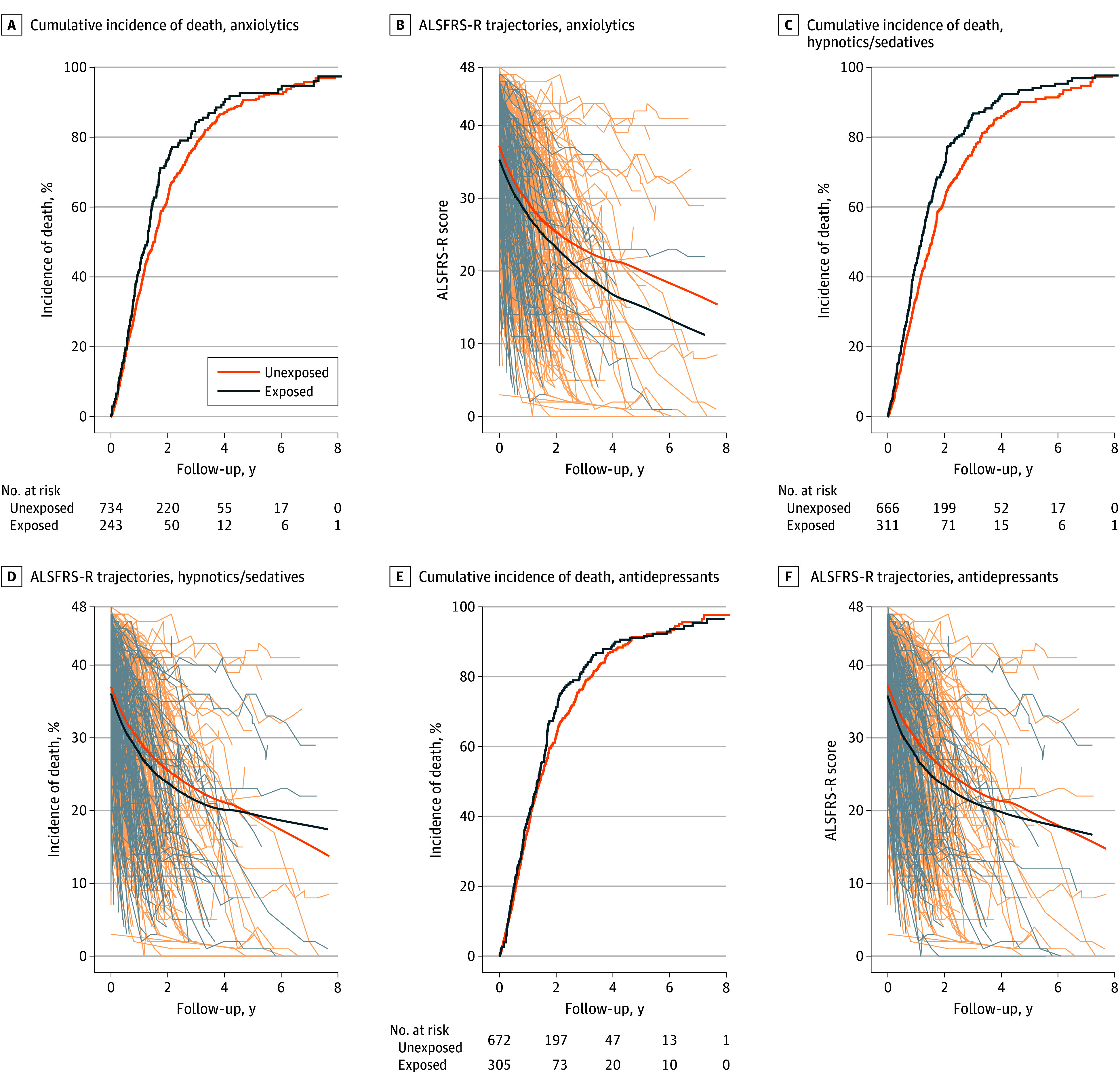
Incidence of Death and Trajectories of Decline Associated With Exposure to Anxiolytics, Hypnotics and Sedatives, and Antidepressants A, C, and E, These plots display the cumulative incidence of death (or use of invasive ventilation) after amyotrophic lateral sclerosis (ALS) diagnosis, comparing patients with ALS who had 2 or more prescriptions of anxiolytics (A), hypnotics and sedatives (C), or antidepressants (E) before diagnosis (exposed) with those without such experience (unexposed). B, D, and F, The spaghetti plots display the individual trajectories of ALS Functional Rating Scale–Revised (ALSFRS-R) score over time since diagnosis, and the mean ALSFRS-R scores, comparing patients exposed and unexposed to the studied medications.

**Table 3.** Risk of Death After Amyotrophic Lateral Sclerosis Diagnosis and Prediagnostic Prescribed Use of Anxiolytics, Hypnotics and Sedatives, or Antidepressants

Psychiatric medication	No. of events (IR)^a^	HR (95% CI)^b^
Anxiolytics	268 (48.58)	1.52 (1.12-2.05)
Hypnotics and sedatives	342 (51.43)	1.23 (0.94-1.61)
Antidepressants	351 (47.62)	1.72 (1.30-2.29)

Abbreviations: HR, hazard ratio; IR, incidence rate.

^a^ IR of death or use of invasive ventilation, per 100 person-years.

^b^ Adjusted for age at diagnosis, sex, body mass index at diagnosis, diagnostic delay, disease progression rate at diagnosis, site of onset, and the time-varying Amyotrophic Lateral Sclerosis Functional Rating Scale–Revised score.

## Discussion

Using data from Swedish nationwide registers, we found that prescribed use of common psychiatric medications was associated with a higher subsequent risk of ALS and a poor survival after ALS diagnosis. In both population and relative comparisons, we found that prescribed use of anxiolytics and antidepressants, ascertained through having at least 2 separate prescriptions during 1 year, 1 to 5 years, or more than 5 years before ALS diagnosis, was associated with a higher risk of ALS (primarily among individuals younger than 65 years). Furthermore, we demonstrated shorter survival and faster functional decline among patients with ALS who had prediagnostic use of psychiatric medications, especially antidepressants, compared with patients without such experience.

Our study showed that prior use of anxiolytics was associated with a higher risk of ALS, in line with previous research showing increased risk of ALS among individuals with a diagnosis of anxiety disorders.^13^ Adaptation to chronic stress has been shown to initially cause anxiety disorders and eventually lead to neurodegeneration.^30,31^ Our study found that prescribed use of antidepressants was associated with a higher risk of ALS, also in agreement with previous studies.^10,12,20^ We found that prior use of anxiolytics or antidepressants was associated with higher mortality risk among ALS patients. Two previous studies^10,11^ found no association between prediagnostic depression or antidepressants use and survival or disease progression, but their reliance on self-reported data limited the comparability with our study. The lower ALSFRS-R score at diagnosis among patients with ALS and prediagnostic use of the studied psychiatric medications might explain this finding to some extent, although we adjusted for it in all models.

Sleep disruption is well documented among patients with ALS^32^ and might be attributed to multifactorial causes, including respiratory dysfunction, muscle cramps, or circadian rhythm disruption.^33,34,35^ As such, sleep disruption might be one of the earliest symptoms for ALS.^36^ Additionally, neuronal damage, reduced neurogenesis, and abnormal level of protein aggregates have been shown to be linked with sleep disruption.^37^ In our study, although most of the hypnotics and sedatives were prescribed with an indication of sleep disturbances, they had also been prescribed for pain (eTable 10 in Supplement 1). Regardless, the higher ALS risk associated with prior use of hypnotics and sedatives was not confirmed in relative comparisons, raising concerns about potential confounding due to shared genetic and environmental factors.

The association between prescribed use of psychiatric medications and ALS risk was noted only among younger individuals (<65 years). This age-specific pattern may be attributed to surveillance bias, health-seeking behaviors, and other factors. Closer monitoring in younger patients with psychiatric symptoms might lead to an earlier ALS diagnosis.^38,39^ Additionally, younger adults tend to seek psychiatric care more frequently, increasing their chances of early ALS detection.^40,41,42^ Finally, psychiatric symptoms might be more prevalent in patients with ALS with an earlier onset, but further validation is needed.

The link between psychiatric medication use during the year before diagnosis and ALS risk likely reflects reverse causation, as psychiatric symptoms might be part of the ALS phenotype or secondary to the severe psychological distress experienced during the diagnostic work-up.^43^ Regardless, increased ALS risk associated with psychiatric medication use more than 5 years before diagnosis might indicate that psychiatric symptoms are prodromal symptoms of ALS as of other neurodegenerative disorders.^44,45,46^ Aggregation of psychiatric disorders in kindreds with ALS might, on the other hand, suggest shared etiopathogenesis between ALS and psychiatric disorders.^16^ Depression, anxiety, and sleep disturbances have been shown to have detrimental effects on neuronal function, resulting in structural brain changes attributed to HPA axis hyperactivity, glutamatergic dysfunction, metabolic shifts, glial alterations, and neuroinflammation.^47,48,49,50,51^ A large register-based study found that antidepressant use was associated with an increased incidence of dementia,^52^ while other studies suggested a neuroprotective effect of antidepressants on the risk of Alzheimer disease.^53,54^ Although corresponding data are scarce in ALS, further studies are needed to differentiate the effect of psychiatric medications from the effect of their indications (ie, underlying psychiatric disorders) on the risk of ALS development. Further adjustment for psychiatric history did not, however, substantially change the results. This lack of difference might be partly attributed to the fact that we were only able to identify a history of psychiatric disorders attended by specialized care, whereas prescribed use of psychiatric medication was ascertained through both specialized and primary care.

A major strength of this study is the availability of extensive clinical data covering the entire follow-up period of all patients with ALS. Additionally, the study benefits from detailed information on prescribed medications, which allowed us to explore temporal associations between previous use of psychiatric medications and ALS risk. The ability to use multiple control groups helped to address potential familial confounding. Finally, we addressed the issue of informative censoring during the follow-up of patients with ALS by modeling the time to death after diagnosis while accounting for the decline in ALSFRS-R scores over time.

### Limitations

This study has limitations. First, the studied medications are prescribed for a wide range of conditions, including depression, anxiety, stress-related disorders, and sleep disorders. Although we had information on the indications for these medications, they overlapped greatly between medications. Second, we used the first prescription of a psychiatric medication as the start of exposure and mapped such exposure to different time windows before ALS diagnosis. However, we had little information on whether the medication was used continuously thereafter, leaving misclassification of the exposure a potential concern. Third, there is a risk for overmatching in the relative comparisons, ie, matching on factors associated with the exposure, and residual confounding, ie, not being able to control for factors not shared between relatives. Fourth, the *C9orf72* variant is strongly linked to behavioral and cognitive changes in ALS.^55^ However, as *C9orf72* variant status was only known for some of the patients with ALS but not the control participants, whether findings of the present study would vary by *C9orf72* variant status awaits investigation. Similarly, whether the higher-than-expected use of psychiatric medications noted among patients with ALS patients is limited to those with specific genetic causes should be examined further. Fifth, we identified use of psychiatric medications through at least 2 prescriptions to increase the specificity of the exposure definition and differentiate temporary use from relatively long-term use. Using at least 1 prescription as the definition of exposure rendered similar results patten, although with smaller magnitude of the associations as expected. Furthermore, 49%, 62%, and 57% of the individuals with exposure to anxiolytics, hypnotics and sedatives, or antidepressants, respectively, had prescriptions made during multiple calendar years before the index date, suggesting a relatively long-term use (data not shown). Finally, the analysis of disease progression in patients with ALS might be more specific to the Stockholm region and the region of western Sweden, as we only included patients with complete data on key survival predictors, such as ALSFRS-R scores and site of onset.

## Conclusions

In this nationwide case-control study, we found that use of anxiolytics, hypnotics and sedatives, or antidepressants was associated with a higher future risk of ALS diagnosis. Prediagnostic use of some of these medications was also associated with shorter survival and faster functional decline among patients with ALS.

## Notes

Supplement 1. **eTable 1.** Adjusted Odds Ratio (OR) With 95% CI of ALS Associated With Prescribed Use of Anxiolytics, Hypnotics and Sedatives, or Antidepressants at 0 to 1, 1 to 5, or More Than 5 Years Before ALS Diagnosis **eTable 2.** Adjusted Odds Ratio (OR) With 95% CI of ALS Associated With Prescribed Use of Anxiolytics, Hypnotics and Sedatives, or Antidepressants More Than 1 or 5 Years Before ALS Diagnosis **eTable 3.** Adjusted Odds Ratio (OR) With 95% CI of ALS Associated With Prescribed Use of Anxiolytics, Hypnotics and Sedatives, or Antidepressants (Defined by at Least 1 Prescription More Than 1 Year Before ALS Diagnosis) **eTable 4.** Adjusted Odds Ratio (OR) With 95% CI of ALS Associated With Prescribed Use of Anxiolytics, Hypnotics and Sedatives, or Antidepressants More Than 1 Year Before ALS Diagnosis **eTable 5.** Adjusted Odds Ratio (OR) With 95% CI of ALS Associated With Prescribed Use of Anxiolytics, Hypnotics and Sedatives, or Antidepressants for Prescriptions During 1 to 2 and 2 to 5 Years Before ALS Diagnosis **eTable 6.** Clinical Characteristics of Patients With ALS With and Without Prediagnostic Prescribed Use of Anxiolytics, Hypnotics and Sedatives, or Antidepressants **eTable 7.** Adjusted Hazard Ratio (HR) With 95% CI for Death After ALS Diagnosis Associated With Prediagnostic Prescribed Use of Anxiolytics, Hypnotics and Sedatives, or Antidepressants Using a Joint Longitudinal-Survival Model After Taking Into Account the Longitudinal Changes of ALSFRS-R in the Time-to-Event Analysis After Stratification by Sex and Age at Diagnosis **eTable 8.** Adjusted Hazard Ratio (HR) With 95% CI for Death After ALS Diagnosis Associated With Prediagnostic Prescribed Use of Anxiolytics, Hypnotics and Sedatives, or Antidepressants Using Cox Model **eTable 9.** Average Change in ALSFRS-R Score over Time (With a Time Unit of Every 6 Months) After ALS Diagnosis Associated With Prediagnostic Prescribed use of Anxiolytics, Hypnotics and Sedatives, or Antidepressants Using Linear Mixed Models With Random Intercept and Slope **eTable 10.** Specific Medications and Reasons for Prescriptions Among Patients With ALS More Than 1 Year Before Diagnosis

Supplement 2.
Data Sharing Statement
