# Supplementary material for: Use of Common Psychiatric Medications and Risk and Prognosis of Amyotrophic Lateral Sclerosis
Source: JAMA Netw Open. 2025 Jun 4;8(6):e2514437. doi: 10.1001/jamanetworkopen.2025.14437 (PMC12138721; doi:10.1001/jamanetworkopen.2025.14437)
Supplement: Supplement 1. — eTable 1. Adjusted Odds Ratio (OR) With 95% CI of ALS Associated With Prescribed Use of Anxiolytics, Hypnotics and Sedatives, or Antidepressants at 0 to 1, 1 to 5, or More Than 5 Years Before ALS Diagnosis eTable 2. Adjusted Odds Ratio (OR) With 95% CI of ALS Associated With Prescribed Use of Anxiolytics, Hypnotics and Sedatives, or Antidepressants More Than 1 or 5 Years Before ALS Diagnosis eTable 3. Adjusted Odds Ratio (OR) With 95% CI of ALS Associated With Prescribed Use of Anxiolytics, Hypnotics and Sedatives, or Antidepressants (Defined by at Least 1 Prescription More Than 1 Year Before ALS Diagnosis) eTable 4. Adjusted Odds Ratio (OR) With 95% CI of ALS Associated With Prescribed Use of Anxiolytics, Hypnotics and Sedatives, or Antidepressants More Than 1 Year Before ALS Diagnosis eTable 5. Adjusted Odds Ratio (OR) With 95% CI of ALS Associated With Prescribed Use of Anxiolytics, Hypnotics and Sedatives, or Antidepressants for Prescriptions During 1 to 2 and 2 to 5 Years Before ALS Diagnosis eTable 6. Clinical Characteristics of Patients With ALS With and Without Prediagnostic Prescribed Use of Anxiolytics, Hypnotics and Sedatives, or Antidepressants eTable 7. Adjusted Hazard Ratio (HR) With 95% CI for Death After ALS Diagnosis Associated With Prediagnostic Prescribed Use of Anxiolytics, Hypnotics and Sedatives, or Antidepressants Using a Joint Longitudinal-Survival Model After Taking Into Account the Longitudinal Changes of ALSFRS-R in the Time-to-Event Analysis After Stratification by Sex and Age at Diagnosis eTable 8. Adjusted Hazard Ratio (HR) With 95% CI for Death After ALS Diagnosis Associated With Prediagnostic Prescribed Use of Anxiolytics, Hypnotics and Sedatives, or Antidepressants Using Cox Model eTable 9. Average Change in ALSFRS-R Score over Time (With a Time Unit of Every 6 Months) After ALS Diagnosis Associated With Prediagnostic Prescribed use of Anxiolytics, Hypnotics and Sedatives, or Antidepressants Using Linear Mixed Models With Random Inte [file jamanetwopen-e2514437-s001.pdf]

Supplemental Online Content

Chourpiliadis C, Lovik A, Ingre C, et al. Use of common psychiatric medications and risk and prognosis of amyotrophic lateral sclerosis. *JAMA Netw Open.* 2025;8(6):e2514437. doi:10.1001/jamanetworkopen.2025.14437

eTable 1. Adjusted Odds Ratio (OR) With 95% CI of ALS Associated With Prescribed Use of Anxiolytics, Hypnotics and Sedatives, or Antidepressants at 0 to 1, 1 to 5, or More Than 5 Years Before ALS Diagnosis

eTable 2. Adjusted Odds Ratio (OR) With 95% CI of ALS Associated With Prescribed Use of Anxiolytics, Hypnotics and Sedatives, or Antidepressants More Than 1 or 5 Years Before ALS Diagnosis

eTable 3. Adjusted Odds Ratio (OR) With 95% CI of ALS Associated With Prescribed Use of Anxiolytics, Hypnotics and Sedatives, or Antidepressants (Defined by at Least 1 Prescription More Than 1 Year Before ALS Diagnosis)

eTable 4. Adjusted Odds Ratio (OR) With 95% CI of ALS Associated With Prescribed Use of Anxiolytics, Hypnotics and Sedatives, or Antidepressants More Than 1 Year Before ALS Diagnosis

eTable 5. Adjusted Odds Ratio (OR) With 95% CI of ALS Associated With Prescribed Use of Anxiolytics, Hypnotics and Sedatives, or Antidepressants for Prescriptions During 1 to 2 and 2 to 5 Years Before ALS Diagnosis

eTable 6. Clinical Characteristics of Patients With ALS With and Without Prediagnostic Prescribed Use of Anxiolytics, Hypnotics and Sedatives, or Antidepressants

eTable 7. Adjusted Hazard Ratio (HR) With 95% CI for Death After ALS Diagnosis Associated With Prediagnostic Prescribed Use of Anxiolytics, Hypnotics and Sedatives, or Antidepressants Using a Joint Longitudinal-Survival Model After Taking Into Account the Longitudinal Changes of ALSFRS-R in the Time-to-Event Analysis After Stratification by Sex and Age at Diagnosis

eTable 8. Adjusted Hazard Ratio (HR) With 95% CI for Death After ALS Diagnosis Associated With Prediagnostic Prescribed Use of Anxiolytics, Hypnotics and Sedatives, or Antidepressants Using Cox Model

eTable 9. Average Change in ALSFRS-R Score over Time (With a Time Unit of Every 6 Months) After ALS Diagnosis Associated With Prediagnostic Prescribed use of Anxiolytics, Hypnotics and Sedatives, or Antidepressants Using Linear Mixed Models With Random Intercept and Slope

eTable 10. Specific Medications and Reasons for Prescriptions Among Patients With ALS More Than 1 Year Before Diagnosis

This supplemental material has been provided by the authors to give readers additional information about their work.

© 2025 Chourpiliadis C et al. *JAMA Network Open*.

eTable 1. Adjusted Odds Ratio (OR) With 95% CI of ALS Associated With Prescribed Use of Anxiolytics, Hypnotics and Sedatives, or Antidepressants at 0 to 1, 1 to 5, or More Than 5 Years Before ALS Diagnosis

Analysis using conditional logistic regression comparing ALS patients with population controls*

	>5 years before ALS diagnosis		1-5 years before ALS diagnosis		0-1 year before ALS diagnosis	
	N. of cases/ controls	OR (95% CI)	N. of cases/ controls	OR (95% CI)	N. of cases/ controls	OR (95% CI)
Anxiolytics	164/732	1.18 (0.98-1.43)	52/146	**1.60 (1.15-2.23)**	62/24	**12.4 (7.44-20.7)**
Hypnotics and sedatives	232/1017	**1.18 (1.00-1.40)**	53/148	**1.46 (1.05-2.03)**	45/29	**6.10 (3.77-9.88)**
Antidepressants	208/906	**1.21 (1.02-1.44)**	64/197	**1.43 (1.06-1.94)**	75/35	**10.1 (6.50-15.7)**

*Adjusted for age at diagnosis, sex, socioeconomic status, educational attainment, and country of birth.

© 2025 Chourpiliadis C et al. *JAMA Network Open*.

eTable 2. Adjusted Odds Ratio (OR) With 95% CI of ALS Associated With Prescribed Use of Anxiolytics, Hypnotics and Sedatives, or Antidepressants More Than 1 or 5 Years Before ALS Diagnosis

Analysis using conditional logistic regression and after stratification by sex and age

	Males	Females	≥65 years	<65 years
**More than 1 year before diagnosis**				
Anxiolytics	**1.42 (1.11- 1.81)**	1.20 (0.96- 1.51)	1.14 (0.93- 1.41)	**1.63 (1.22- 2.18)**
Hypnotics and sedatives	**1.34 (1.07- 1.68)**	1.19 (0.96- 1.47)	1.04 (0.86- 1.25)	**1.82 (1.40- 2.38)**
Antidepressants	**1.46 (1.16- 1.84)**	1.19 (0.96- 1.47)	1.15 (0.95- 1.40)	**1.67 (1.27- 2.18)**
**More than 5 years before diagnosis**				
Anxiolytics	1.26 (0.95- 1.67)	1.16 (0.90- 1.49)	1.01 (0.80- 1.27)	**1.66 (1.22- 2.26)**
Hypnotics and sedatives	1.27 (0.99- 1.62)	1.13 (0.91- 1.41)	0.99 (0.81- 1.21)	**1.77 (1.32- 2.37)**
Antidepressants	**1.34 (1.04- 1.74)**	1.12 (0.89- 1.41)	1.07 (0.86- 1.32)	**1.58 (1.18- 2.12)**

*Adjusted for age at diagnosis, sex, socioeconomic status, educational attainment, and country of birth.

© 2025 Chourpiliadis C et al. *JAMA Network Open*.

eTable 3. Adjusted Odds Ratio (OR) With 95% CI of ALS Associated With Prescribed Use of Anxiolytics, Hypnotics and Sedatives, or Antidepressants (Defined by at Least 1 Prescription More Than 1 Year Before ALS Diagnosis)

Analysis of the population comparison using conditional logistic regression*

Psychiatric medications	No. of cases/ controls	OR (95% CI)
Anxiolytics	234/934	1.18 (0.99- 1.39)
Hypnotics and sedatives	277/1119	1.14 (0.97- 1.34)
Antidepressants	255/1054	1.13 (0.96- 1.33)
*Conditioned on age, sex, and calendar time, and adjusted for socioeconomic status, educational attainment, and country of birth.		

© 2025 Chourpiliadis C et al. *JAMA Network Open*.

eTable 4. Adjusted Odds Ratio (OR) With 95% CI of ALS Associated With Prescribed Use of Anxiolytics, Hypnotics and Sedatives, or Antidepressants More Than 1 Year Before ALS Diagnosis

Analysis using conditional logistic regression and further adjusting for any previous psychiatric diagnoses.

	Prescribed use up to 1 year before ALS diagnosis	
Psychiatric conditions	Model 1*	Model 2*
Anxiolytics	1.34 (1.12- 1.60)	1.36 (1.13- 1.64)
Hypnotics and sedatives	1.21 (1.02- 1.43)	1.22 (1.03-1.45)
Antidepressants	1.26 (1.06- 1.49)	1.28(1.08- 1.53)

Model 1 is adjusted for sex, year of birth, socioeconomic status, education and country of birth
Model 2 is adjusted for sex, year of birth, socioeconomic status, education, country of birth, and history of any psychiatric disorder.

© 2025 Chourpiliadis C et al. *JAMA Network Open*.

eTable 5. Adjusted Odds Ratio (OR) With 95% CI of ALS Associated With Prescribed Use of Anxiolytics, Hypnotics and Sedatives, or Antidepressants for Prescriptions During 1 to 2 and 2 to 5 Years Before ALS Diagnosis

	2-5 years before ALS diagnosis		1-2 years before ALS diagnosis	
	N. of cases/ controls	OR (95% CI)	N. of cases/ controls	OR (95% CI)
Anxiolytics	35/114	1.35 (0.91-2.00)	17/28	2.73 (1.47-5.05)
Hypnotics and sedatives	31/116	1.07 (0.71-1.61)	22/30	2.93 (1.66-5.17)
Antidepressants	39/153	1.10 (0.76-1.59)	25/38	2.56 (1.53-4.29)
*Adjusted for age at diagnosis, sex, socioeconomic status, educational attainment, and country of birth.				

© 2025 Chourpiliadis C et al. *JAMA Network Open*.

eTable 6. Clinical Characteristics of Patients With ALS With and Without Prediagnostic Prescribed Use of Anxiolytics, Hypnotics and Sedatives, or Antidepressants

	**Pre-diagnostic prescribed use of psychiatric medications**		
**Characteristics**	**No (N=488)**	**Yes (N=569)**	**P for difference**
**Male, N (%)**	291 (59.6%)	270 (47.5%)	**<0.001**
**Age at diagnosis, mean (SD)**	67.2 (11.7)	67.8 (11.4)	0.5
**BMI at diagnosis, mean (SD)**	23.5 (4.2)	24.0 (4.4)	0.2
**Diagnostic delay in months, median (p25-p75)**	12.1 (7.7, 17.7)	12.1 (7.4, 17.1)	0.9
**Bulbar onset**	101 (20.7%)	129 (22.6%)	0.6
**Familial ALS, N (%)**	28 (5.7%)	21 (3.7%)	0.1
**ALSFRS-R at diagnosis, mean (SD)**	37.1 (7.6)	35.2 (8.4)	**0.003**
**Gastrostomy, N (%)**	107 (21.9%)	132 (23.2%)	0.6
**Invasive ventilation, N (%)**	9 (1.8%)	7 (1.2%)	0.4
**Dementia, N (%)**	29 (5.9%)	45 (7.9%)	0.4
**Death or invasive ventilation, N (%)**			
**Age at death, mean (SD)**	70.4 (9.4)	70.3 (10.1)	0.9

BMI: body mass index (Kg/m2), SD: standard deviation

© 2025 Chourpiliadis C et al. *JAMA Network Open*.

eTable 7. Adjusted Hazard Ratio (HR) With 95% CI for Death After ALS Diagnosis Associated With Prediagnostic Prescribed Use of Anxiolytics, Hypnotics and Sedatives, or Antidepressants Using a Joint Longitudinal-Survival Model After Taking Into Account the Longitudinal Changes of ALSFRS-R in the Time-to-Event Analysis After Stratification by Sex and Age at Diagnosis

Pyschiatric medications	Males	Females	≥65 years	<65 years
Anxiolytics	1.58 (0.95- 2.63)	1.46 (0.96- 2.23)	**1.60 (1.08- 2.36)**	1.53 (0.86- 2.72)
Hypnotics and sedatives	1.12 (0.72- 1.73)	1.31 (0.90- 1.91)	1.11 (0.79- 1.57)	1.31 (0.78- 2.19)
Antidepressants	**1.98 (1.21- 3.25)**	**1.76 (1.21- 2.57)**	**1.70 (1.18- 2.43)**	**1.99 (1.18- 3.35)**

IR: incidence rate of death or use of invasive ventilation, per 100 person-years.
*Adjusted for age at diagnosis, sex, BMI at diagnosis, diagnostic delay, disease progression rate at diagnosis, site of onset, and the time-varying ALSFRS-R.

© 2025 Chourpiliadis C et al. *JAMA Network Open*.

eTable 8. Adjusted Hazard Ratio (HR) With 95% CI for Death After ALS Diagnosis Associated With Prediagnostic Prescribed Use of Anxiolytics, Hypnotics and Sedatives, or Antidepressants Using Cox Model*

Psychiatric medications	Number of events (IR)	HR (95% CI)
Anxiolytics	199 (56.39)	**1.34 (1.02- 1.77)**
Hypnotics and sedatives	261 (58.70)	1.22 (0.94- 1.57)
Antidepressants	250 (52.37)	**1.48 (1.13- 1.93)**

IR: incidence rate of death or use of invasive ventilation, per 100 person-years.

*Adjusted for age at diagnosis, sex, BMI at diagnosis, diagnostic delay, disease progression rate at diagnosis, site of onset, and ALSFRS-R score measured at diagnosis.

© 2025 Chourpiliadis C et al. *JAMA Network Open*.

eTable 9. Average Change in ALSFRS-R Score over Time (With a Time Unit of Every 6 Months) After ALS Diagnosis Associated With Prediagnostic Prescribed use of Anxiolytics, Hypnotics and Sedatives, or Antidepressants Using Linear Mixed Models With Random Intercept and Slope

Psychiatric medications	β coefficient (95% CI)*	β coefficient (95% CI)†
Anxiolytics	-1.42 (-3.49, 0.66)	-1.47 (-3.55, 0.61)
Hypnotics and sedatives	-0.81 (-2.76, 1.13)	-0.80 (-2.74, 1.14)
Antidepressants	**-2.48 (-4.41, -0.55)**	**-2.50 (-4.44, -0.57)**

*Adjusted for age at diagnosis, sex, BMI at diagnosis, diagnostic delay, and site of onset.
†Additionally adjusted for ALSFRS-R score measured at diagnosis.

© 2025 Chourpiliadis C et al. *JAMA Network Open*.

eTable 10. Specific Medications and Reasons for Prescriptions Among Patients With ALS More Than 1 Year Before Diagnosis

ATC	Drug name	Reason for prescription
Anxiolytics		
N05BA	Diazepam, Stesolid, Sobril, Oxascand, Alprazolam, Xanor	Symptoms of anxiety<br>Panic attack<br>Symptoms of depression<br>Sleep disturbances<br>Muscle tension<br>Restless legs<br>Analgesic
N05BB	Atarax, Hydroxizin	Symptoms of anxiety<br>Sleep disturbances<br>Allergies<br>Pruritus
Hypnotics and sedatives		
N05CD	Apodorm, Nitrazepam, Flunitrazepam, Triazolam, Halcion	Sleep disturbances
N05CF	Imovane, Zopiclone, Zolpidem, Stilnoct, Sonata	Sleep disturbances
N05CH	Circadin	Sleep disturbances
N05CM	Heminevrin, Propavan	Sleep disturbances<br>Pain
Antidepressives		
N06AA	Anafranil, Klomipramin, Amitryptilin, Saroten, Tryptizol	Pain<br>Sleep disturbances<br>Symptoms of depression
N06AB	Fluoxetin, Cipramil, Citalopram, Paroxetin, Seroxat, Oralin, Sertralin, Zoloft, Cipralex, Escitalopram	Symptoms of anxiety<br>Depression<br>Pain
N06AX	Mirtazapin, Remeron, Voxra, Zyban, Efexor, Venlafaxin, Edronax, Cymbalta, Duloxetin	Symptoms of anxiety<br>Depression<br>Pain<br>Nicotine dependance<br>Sleep disturbances

© 2025 Chourpiliadis C et al. *JAMA Network Open*.
