# Supplementary material for: Use of Common Psychiatric Medications and Risk and Prognosis of Amyotrophic Lateral Sclerosis
Source: JAMA Netw Open. 2025 Jun 4;8(6):e2514437. doi: 10.1001/jamanetworkopen.2025.14437 (PMC12138721; doi:10.1001/jamanetworkopen.2025.14437)
Supplement: Supplement 2. — Data Sharing Statement [file jamanetwopen-e2514437-s002.pdf]

Data Sharing Statement

Chourpiliadis. Use of Common Psychiatric Medications and Risk and Prognosis of Amyotrophic Lateral Sclerosis. *JAMA Netw Open*. Published June 04, 2025.
doi:10.1001/jamanetworkopen.2025.14437

Data
Data available: No
